# ORMEF: a Mediterranean database of exotic fish records

**DOI:** 10.1038/s41597-022-01487-z

**Published:** 2022-06-25

**Authors:** Ernesto Azzurro, Sonia Smeraldo, Annalisa Minelli, Manuela D’Amen

**Affiliations:** 1 CNR-IRBIM. National Research Council. Institute of Biological Resources and Marine Biotechnologies, Ancona, Italy; 2 Stazione Zoologica Anton Dohrn, Naples, Italy; 3 Stazione Zoologica Anton Dohrn, Fano Marine Centre, Fano, PU Italy; 4 ISPRA. Institute for Environmental Protection and Research, DG-SINA, Rome, Italy; 5 ISPRA. Institute for Environmental Protection and Research, PRES-PSMA, Rome, Italy

**Keywords:** Ichthyology, Marine biology, Invasive species

## Abstract

The Mediterranean Sea is recognized today as the World’s most invaded marine region, but observations of species occurrences remain scattered in the scientific literature and scarcely accessible. Here we introduce the ORMEF database: a first comprehensive and robust compilation of exotic fish observations recorded over more than a century in the Mediterranean. ORMEF consists today of 4015 geo-referenced occurrences from 20 Mediterranean Countries, extracted from 670 scientific published papers. We collated information on 188 fish taxa that are thus divided: 106 species entered through the Suez Canal; 25 species introduced by shipping, mariculture, aquarium release or by means of other human activities; 57 Atlantic species, whose arrival in the Mediterranean has been attributed to the unassisted immigration through the strait of Gibraltar. Each observation included in the ORMEF database was submitted to a severe quality control and checked for geographical and taxonomic biases. ORMEF is a new authoritative reference for Mediterranean bio-invasion research and a living archive to inform management strategies and policymakers in a period of rapid environmental transformation.

## Abstract (summary)

**Table:** 

Measurement(s)	non-indigenous species’ name • AphiaID code • non-indigenous species’ family • observations’ category • year of observation • country • geographical precision of coordinates • latitude • longitude
Technology Type(s)	observation • classification
Factor Type(s)	Species • Family • Category • Year • Country • Precision of coordinates • Decimal_lat • Decimal_long • Source
Sample Characteristic - Organism	Fishes
Sample Characteristic - Environment	marine environment
Sample Characteristic - Location	Mediterranean Sea

## Background & Summary

Maritime traffic, mariculture, aquarium trade and above all, entries through the Suez Canal made the Mediterranean one of the most invaded marine regions in the world^1,2^. A large number of non-indigenous species (NIS) has been already introduced to this basin^3–5^, producing a variety of ecological and socio-economic impacts^6^. The Mediterranean is also warming faster than any other marine region^7,8^, becoming increasingly suitable to be invaded by organisms of tropical origin. Among other non indigenous taxa, fish species provide the best documented and impressive examples of this phenomenon^9^, with increasing efforts dedicated to monitor their occurrence and progressive expansion^10^.

In the last decades, several databases on invasive species have been implemented, such as AquaNIS (www.corpi.ku.lt/databases/aquanis)^11^, DAISIE (https://www.gbif.org/dataset/39f36f10-559b-427f-8c86-2d28afff68ca)^12^, EASIN (https://easin.jrc.ec.europa.eu/)^13^, ESENIAS (http://www.esenias.org/)^14^, ELNAIS (https://www.eea.europa.eu/data-and-maps/data/external/elnais-invasive-alien-species-data)^15^, NOBANIS (https://www.nobanis.org/)^16^, MAMIAS (http://www.mamias.org/)^17^, (MedMIS. http://www.iucn-medmis.org)^18^, some under the promotion of the European Union, but they often lack regular updates and may suffer of several biases that limit their usefulness for delivering timely and reliable information^5^. Most importantly, most of these databases only provide information for large geographic-subsectors, with no georeferenced information at the level of single observations. Similarly, several lists of NIS have been published in Mediterranean literature^5,19–27^, but most occurrence data remain hidden and widely dispersed in the scientific literature.

Here we introduce the ORMEF (Occurrence Records of Mediterranean Exotic Fishes) database, as a first comprehensive, harmonized, and robust compilation of ‘exotic’ fish occurrences in the Mediterranean Sea. We deliberately used the term ‘exotic’ in quotes since our dataset includes not only NIS that are introduced by human activities but it is also extended to Atlantic fishes that are presumably arrived through the straits of Gibraltar without the direct assistance of human agency. Considering the mostly adopted definition of the terms exotic alien or NIS^28,29^, this latter group of *neonative* species (*sensu* Essl *et al*.^30^) cannot be considered as such. Nevertheless, their inclusion in the ORMEF database is motivated by two important considerations: first, scientific evidences about the introduction means are typically lacking or weak in the Mediterranean literature, and for many of these species we cannot completely discard the hypothesis of a possible introduction by human activities; second, Atlantic fishes entering the Mediterranean through the straits of Gibraltar, have been considered as ‘exotic’ in previous Mediterranean inventories^10,31^, and their occurrences in the Mediterranean basin are worth to be closely traced.

## Methods

Occurrence records were gathered through an extensive literature search, updating and implementing a previous version of the ORMEF database, that had previously been employed for large scale investigations on invasive fishes^2,9,30^. This offline database, once limited to the most successful fish invaders of the Mediterranean, is here extended to presumably all the non indigenous and neonative fishes recorded so far in this region, up to the most recently documented introductions.

### Literature data extraction

Literature search was performed mainly through Google Scholar (https://scholar.google.com/), ISI Web of Science (https://www.webofscience.com/), and Scopus (https://www.scopus.com/), by multiple search criteria and using the scientific names of the species and a combination of terms such as exotic, non-indigenous, alien in conjunction with the names of Mediterranean and/or Mediterranean countries, in the title, abstract, and keywords. In addition, we periodically checked the main journals devoted to the publication of exotic fish records to periodically update the database with new georeferenced occurrences. Grey literature was also considered, when accessible. All the historical observations of species are considered, from the earliest documented records to the most recent ones included in the latest version of ORMEF (October 2020), which extracts data from 670 papers published between 1902–2020^32^.

### Dataset final collation

Each record extracted from the scientific literature, was associated with the name of the species, year of detection, presumed introduction path, and the country where the species was observed. Also the bibliographic references, representing the source of each georeferenced record, are reported in the database.

The list of species included in the ORMEF database follows the authoritative CIESM Atlas of exotic species^10^, adopting the same terminology. In agreement with this atlas, we grouped the species according to their presumed introduction path: EXOTIC CAN = fishes introduced through the Suez Canal; EXOTIC HM = fishes introduced by other human vectors, such as shipping, mariculture or aquarium release; NRE (natural range expansion) = fishes of Atlantic origin, which are supposed to have entered into the Mediterranean through Gibraltar, without direct assistance of human agency. Thus the term ‘natural’ would indicate that the presumed vector is not anthropogenic.

The ORMEF database is currently enriched with the most recent information on new arrivals, range expansions, changes in abundances, changes in identification/nomenclature/taxonomy. Each georeferenced string included in ORMEF was submitted to a severe quality control and checked for possible geographical and taxonomic biases. All records were manually verified to identify potential outliers and in-land data points. These records were checked against the information provided by the original source and manually moved to the localities indicated in the source, only when wrongly reported.

For those published records missing coordinates, Latitude and Longitude were manually derived from Google Earth (https://earth.google.com/web/) based on geographical information reported in the original source, such as the name of record location, the distance from the coasts and the depth. Duplicate records were removed.

## Data Records

### General consideration

Once subjected to the quality control procedures, the final dataset consisted of 4015 georeferenced records of occurrence on 188 accepted species of fish, and 83 families. It is publicly accessible for download from SEANOE, a permanent repository hosting sea-related open data (10.17882/84182)^33^, and it follows the FAIR principle of Findability, Accessibility, Interoperability and Reusability of data^34^.

The dataset structure was based on Darwin Core Standard (DwC, https://dwc.tdwg.org/), and taxonomic information was extracted from the World Register of Marine Species (WoRMS; www.marinespecies.org). This tool provides a unique identifier (aphiaID) that was added to the ORMEF database, linking each taxon to an internationally accepted standardized name with associated taxonomic information (including hierarchy, rank, acceptance status and synonymy) that will continue to be updated with respect to any possible taxonomic changes that could happen in the future.

As already described, species were assigned to three different groups (EXOTIC CAN, EXOTIC HM and NRE), depending on their entry mode. Each observation was associated with information on the Year and Country of the sighting and complemented with geographical coordinates expressed as decimal degrees and according to three different levels of precision: *Pre* = Precise (radius of ≤1 km); *App* = Approximate (radius of >1 km and ≤10 km); *Con* = Conventional (radius >10 km). Each reported sighting was associated with its respective literature source including permanent identifiers (bibliographic reference, with DOI) when available. Overall, 12 fields were associated with each record (Table 1).

**Table 1:** Database fields used by ORMEF.

Field	Description
RecordID	A progressive code univocally identifying each record.
Species	Scientific name of the species, according to Fisher *et al*., 2019
AphiaID	Unique identifier of the species provided by the World Register of Marine Species (WoRMS; www.marinespecies.org).
Family	Family taxonomic rank.
Category	Path used by the species to reach the Mediterranean Sea.
Year	The four-digit year in which the record occurred.
Country	Country in which the record occurred.
Precision of coordinates	Pre = Precise (radius of ≤1 km); App = Approximate (radius of >1 km and ≤10 km); Con = Conventional (radius >10 km).
decimalLatitude	Geographical latitude in decimal degrees of the record location.
decimalLongitude	Geographical longitude in decimal degrees of the record location.
Source	The source of the record. The name of the author and the publication date is provided. For sources with more than two authors the abbreviation “*et al*.” is used.
DOI	Digital Object Identifier of the source, where present.

### Spatial and temporal coverage

**Fig. 1:**
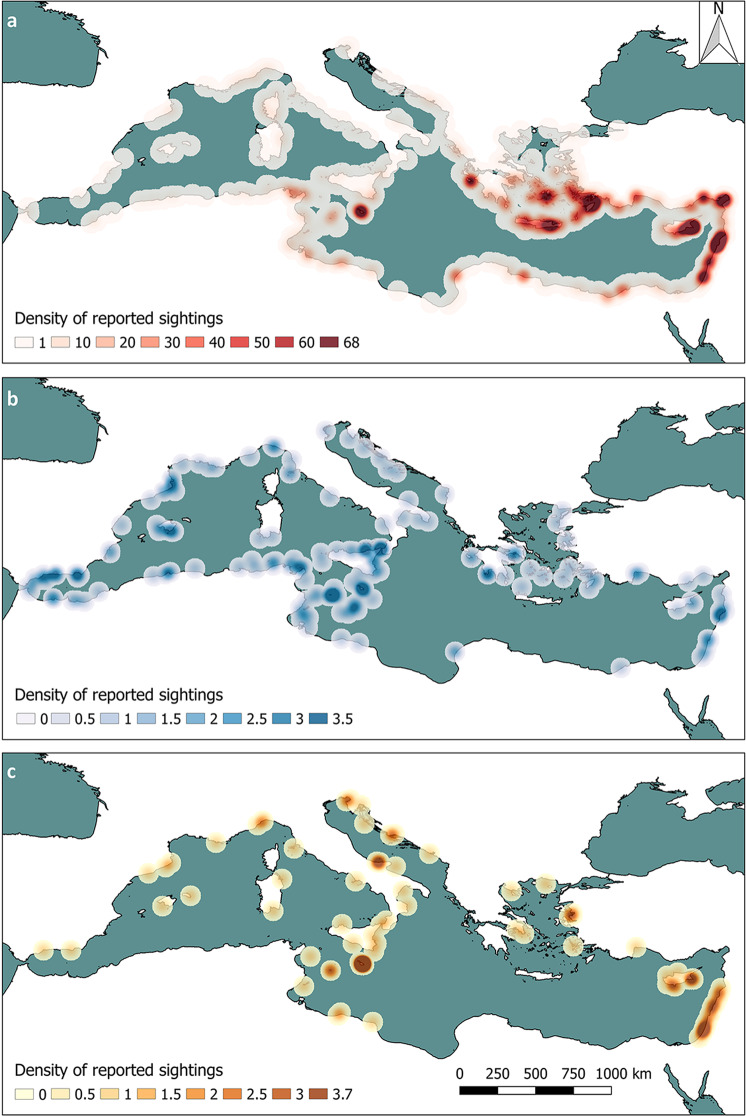
Heat maps of the occurrences of non-indigenous species. Cumulative density of reported sightings (radius = 70 km) for (**a**) EXOTIC CAN, (**b**) NRE and (**c**) EXOTIC HM species in the Mediterranean Sea.

**Fig. 2:**
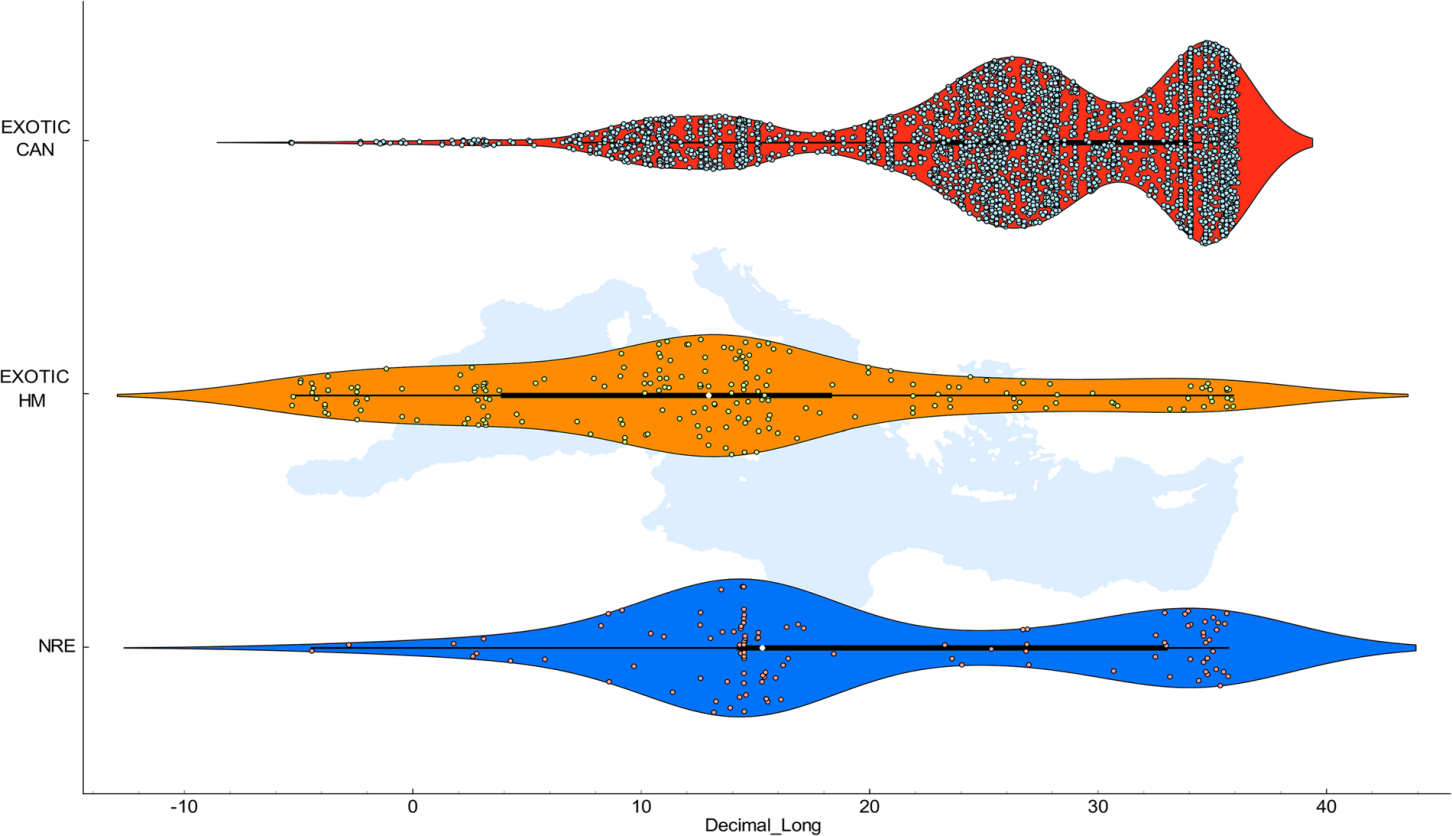
Geographical distribution of the data along the Longitudinal axis. For each group EXOTIC CAN, EXOTIC HM and NRE, the violin plots show the kernel probability density of the occurrence data and include a box indicating the interquartile range of the data with the white marker indicating their median value. Real records are represented within the violin shape with dots.

**Fig. 3:**
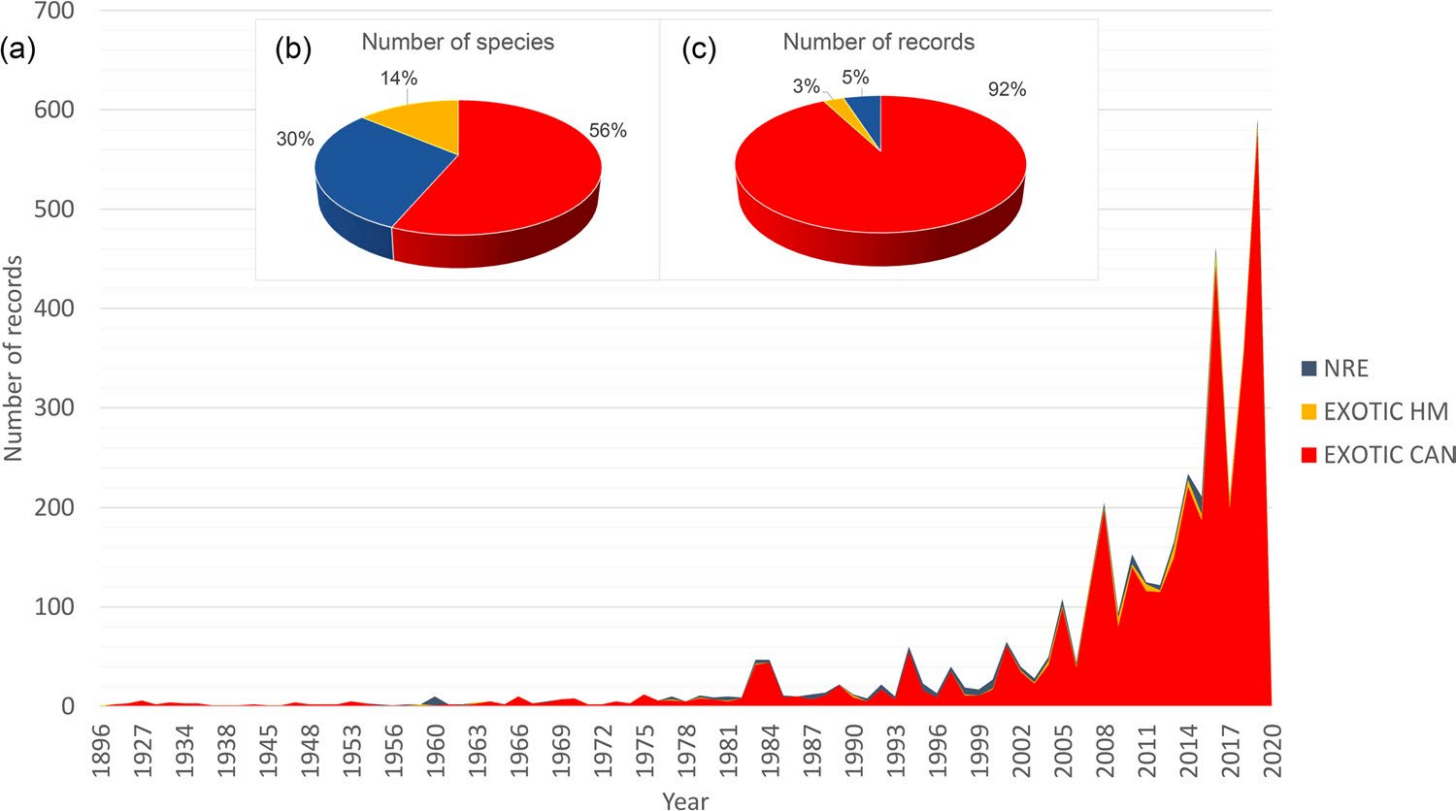
Temporal repartition of data among the groups. (**a**) Cumulative number of records along the temporal axis for the three groups EXOTIC CAN, EXOTIC HM and NRE. The Pie Charts report the proportion number of (**b**) species and (**c**) records for each of the above-mentioned groups.

The records were distributed in 20 different countries, all over the Mediterranean Region, between the years 1896 and 2020. Geographical distribution of the data, according to the three main groups of species is given in Fig. 1. A clear geographical pattern is visible only for EXOTIC CAN, whose distribution of records is strongly skewed toward the East (Figs. 1 and 2). On the contrary, no clear geographic pattern is apparent for EXOTIC HM and NRE (Figs. 1 and 2). The distribution of records is uneven among the different Mediterranean countries (Table 2) with Greece, Turkey, Cyprus, and Lebanon accounting for the 65% of the observations (and 36% of species) registered so far in the Mediterranean Sea. The overall number of records per year follows an exponential growth and is dominated by EXOTIC CAN, which is far more reported with respect to EXOTIC HM and NRE (Fig. 3).

**Table 2:** Number of Records, Species and Families according to each Country.

Country	Records	Species	Families	Max Year	Min Year
Albania	7	4	4	2015	1986
Algeria	44	10	10	2019	1955
Croatia	25	13	11	2016	1896
Cyprus	381	40	29	2019	1929
Egypt	146	54	37	2019	1902
France	51	7	7	2019	1980
Greece	1298	49	36	2019	1934
Israel	201	99	58	2019	1927
Italy	247	40	31	2020	1958
Lebanon	347	59	43	2020	1962
Libya	183	31	22	2019	1966
Malta	147	27	17	2019	1993
Montenegro	6	4	4	2016	2000
Morocco	8	4	4	2018	1960
Palestine	3	3	3	2019	2018
Slovenia	4	3	3	2013	2007
Spain	71	27	16	2019	1977
Syria	41	29	23	2019	1929
Tunisia	197	41	32	2020	1960
Turkey	608	78	53	2020	1942

Countries are listed in decreasing order, according to the number of records. The highest number of records, species and families is in bold. For each Country, the year of older and the year of the latest record are indicated with ‘Min Year’ and ‘Max Year’, respectively.

## Technical Validation

In agreement with Golani *et al*.^10^, we excluded questionable, cryptogenic, brackish, and vagrant species from our list of taxa. Species names were checked with Fricke *et al*. (2021) (https://www.calacademy.org/scientists/projects/eschmeyers-catalog-of-fishes)^35^ taking into account recent taxonomic changes and documented misidentifications^36,37^.

Only records identified at the species level were kept into the database, whilst genus level identifications, including the ones of *Abudefduf* spp^38^. were not considered.

## Usage Notes

The ORMEF database is presented here as the most accurate source of information on the distribution of non-indigenous and neonative fishes in the Mediterranean Sea and it is publicly accessible for download in a SEANOE repository^33^. The dataset comes with the complete list of references from which data has been extracted. ORMEF represents an authoritative geo-referenced dataset to serve various needs of bioinvasion research, such as Species Distribution Modelling, invasion dynamics, speed rate calculations, and future comparison in the Mediterranean area and beyond. ORMEF can be also considered as a novel authoritative source of information for regional monitoring programs, mainly the Marine Strategy Framework Directive of the European Union, and the Integrated Monitoring and Assessment Programme of the Mediterranean Sea and Coast and related Assessment Criteria^39^. Data can be also used to highlight changes in the monitoring effort through time and among the different Mediterranean countries. It should be noted that ORMEF does not consider non georeferenced checklists and thus it is advisable to integrate this information when compiling or updating inventories at the level of countries or Mediterranean subregions.

In the future, ORMEF will be subjected to periodical updates and implemented with new fields of information, which may further expand the applications of this dataset to predict and to map future species distribution according to climate change scenarios.

## Data Availability

No custom code was used to generate or process the data described in this manuscript.
